# Hydrogels for Bone Repair: Construction Strategies and Applications

**DOI:** 10.1002/smmd.70036

**Published:** 2026-04-29

**Authors:** Miaomiao Wang, Qianwei Su, Yuezhou Wu, Xiaoyi Liang, Fengjin Zhou, Yingying Jing, Zhanghua Li, Jiacan Su

**Affiliations:** ^1^ Institute of Translational Medicine Shanghai University Shanghai China; ^2^ Department of Rehabilitation Medicine Shanghai Zhongye Hospital Shanghai China; ^3^ MedEng‐X Institutes Shanghai University Shanghai China; ^4^ Department of Polymer Science and Engineering ERC of Membrane Water Treatment (MOE), Key Laboratory of Macromolecular Synthesis and Functionalization (MOE) Zhejiang University Hangzhou China; ^5^ Department of Orthopedics Xinhua Hospital Affiliated to Shanghai Jiao Tong University School of Medicine Shanghai China; ^6^ Department of Orthopedics Honghui Hospital Xi'an Jiao Tong University Xi'an China; ^7^ National Center for Translational Medicine SHU Branch Shanghai University Shanghai China; ^8^ School of Nursing and Health Management Shanghai University of Medicine and Health Sciences Shanghai China; ^9^ Department of Orthopedics Wuhan Third Hospital, Tongren Hospital of Wuhan University Wuhan China

**Keywords:** angiogenesis, bone regeneration, hydrogel, osteoimmunomodulation, smart‐responsive

## Abstract

Bone injuries, particularly those associated with an aging global population, pose a persistent and complex clinical challenge. Current gold‐standard treatments such as autografts, allografts, and metal implants, are often limited by immune rejection and mechanical mismatch. In this context, hydrogels are promising biomaterials for bone regeneration, due not only to inherent biocompatibility, high hydration, and tunable elasticity but also to the ability to mimic the native bone micro environment. This review presents a critical and comprehensive analysis of how hydrogels for bone repair are designed, constructed, and functionalized to achieve the desired regenerative performance. It systematically examines multiple hydrogel types, centering on the design of their strength and biodegradation features to better align with the functional demands of bone healing. Furthermore, the review summarizes that by incorporating bioactive molecules, nanomaterials, or cells, advanced functionalization can orchestrate osteogenesis and angiogenesis. In vitro and in vivo studies evidenced the performance of hydrogels' applications ranging from bone fracture repair to smart, stimuli‐responsive platforms for personalized regenerative medicine. This review finally identifies the prevailing translational challenges and suggests future research trajectories.

## Key Points

- This review establishes an integrated framework linking the pathophysiology of bone repair to the design criteria of bone‐regenerative hydrogels, including biocompatibility, extracellular matrix (ECM) mimicry, tunable mechanics, and programmable biodegradation.
- This review summarizes representative applications spanning bone fracture repair, critical‐sized defects, and smart stimuli‐responsive platforms, and delineates key translational barriers to accelerate clinical implementation of hydrogel‐based therapies.

## Graphical abstract

This review establishes an integrated framework for bone repair hydrogels, covering construction strategies from material selection to functional modification, mechanical and biological characterization, and diverse applications including defect repair, fracture healing, and smart responsive systems.

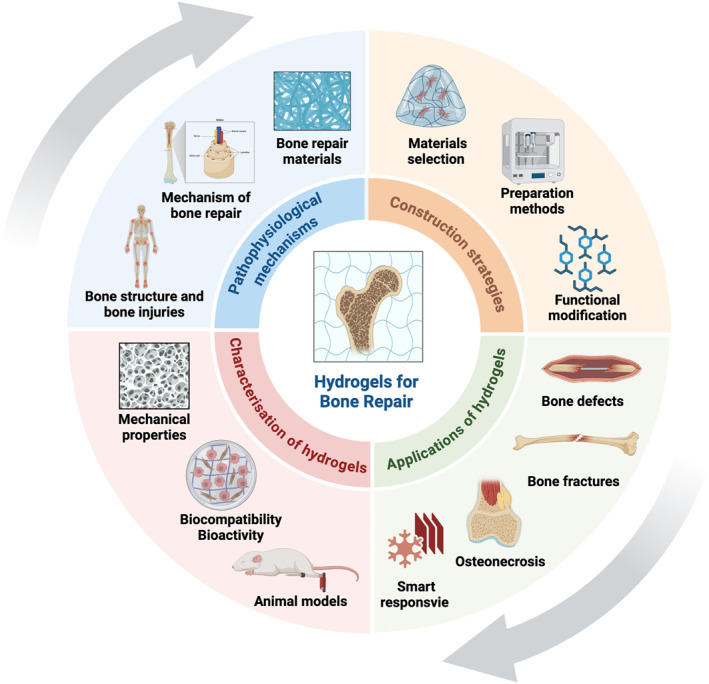

## 1 Introduction

The management of bone diseases (including osteoporosis, osteoarthritis, traumatic fractures, osteonecrosis, and bone tumors) is still a great challenge in clinical practice. These diseases are caused by a variety of causes such as genetic susceptibility, metabolic imbalance, trauma and degeneration process, which usually exceed the body's inherent regeneration ability [1]. Osteoporosis alone affects about 200 million people around the world, and the associated brittle fractures constitute a huge and growing burden. It is expected that by 2050, hip fractures caused by osteoporosis worldwide will double [2, 3]. This rising prevalence highlights the urgent need for more effective treatment interventions, especially for patients whose natural healing process is impaired or inadequate [4]. Traditional therapies based on plants (autologous, allogeneic, heterologous transplants) are still limited by obvious shortcomings, such as the incidence of acquisition sites, insufficient donor resources, the spread of infectious factors and rejection driven by host immunity [5]. Therefore, the development of new and complex bone repair materials has become the top priority of orthopedic research and regenerative medicine.

Hydrogel is a polymer material with a hydrophilic three‐dimensional (3D) structure, which makes it have a unique set of properties [6]. Their composition comes from natural or synthetic precursors, which can be precisely customized by various cross‐linking strategies to achieve the required mechanical strength and biocompatibility [7, 8]. This tunability is the core of its use for bone repair [9]. Its high hydration level and conformability enable it to simulate bionic bone extracellular matrix (ECM), which helps to reduce the inflammatory response after implantation [10]. Injectable and other functions make minimally invasive delivery possible and adaptive filling complex defect geometry, while reducing surgical trauma and improving patient recovery [11]. Their biodegradable scaffold structure ensures that they provide temporary support without surgical resection and are gradually replaced by neonatal bone tissue [12].

This review is structured to critically evaluate the evolving paradigm of hydrogel‐based strategies for bone regeneration. It outlines the pathophysiological context of bone repair and establishes the biological requirements that these materials must meet. The core of our discussion delves into the material selection and sophisticated construction strategies employed to create functional hydrogels, with a critical analysis of how different design choices impact their performance. The review then systematically examines the application of these engineered hydrogels across various bone repair scenarios, from critical‐sized defects to complex fracture non‐unions.

**FIGURE 1:**
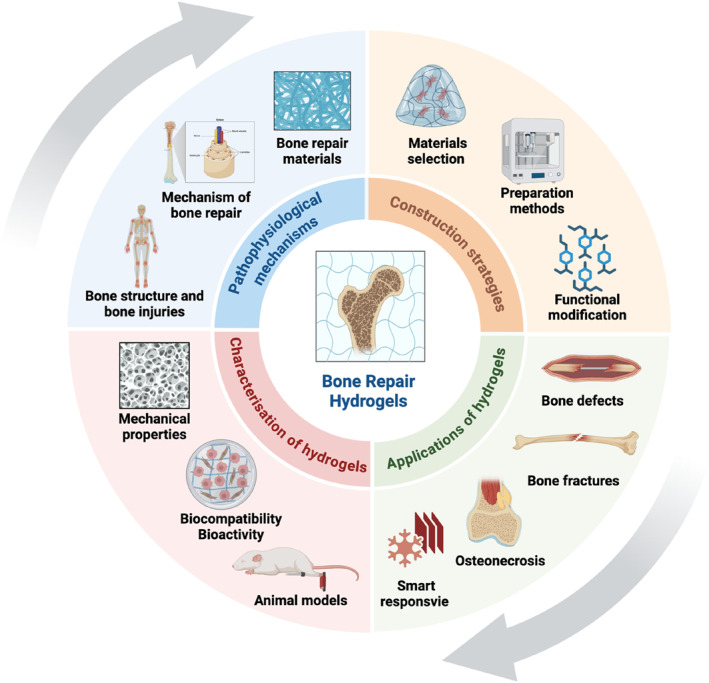
Design and translational pathway of hydrogels for bone repair. An integrated framework of mechanisms, construction, characterization, and applications. Created with BioRender.com.

This review critically evaluates the evolutionary paradigm of bone regeneration strategies based on hydrogels. It outlines the pathophysiological background of bone repair and establishes the biological requirements that these materials must meet. The core of discussion is the material selection and complex construction strategies used to create functional hydrogels, and critically analyze how different design options affect their performance. This review introduces the application of these engineering hydrogels in various bone repair scenarios, from critical‐sized defects to complex fracture non‐healing. This review also puts forward the ongoing challenges of hindering the clinical transformation of bone repair, and puts forward insights into the future research direction of hydrogel for bone repair (Figure 1).

## 2 Pathophysiological Mechanisms of Bone Repair

**FIGURE 2:**
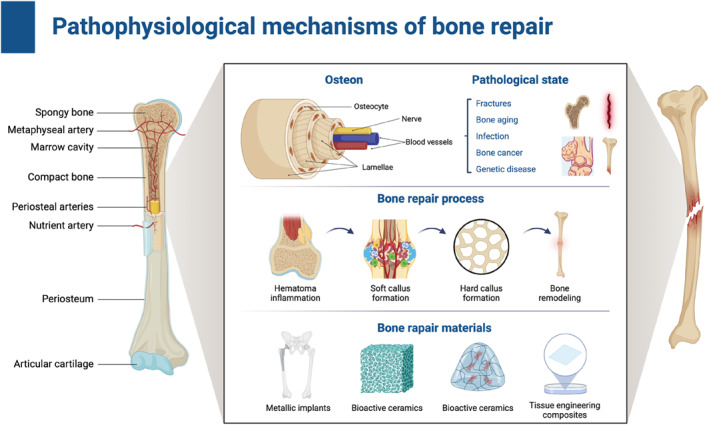
Pathophysiological mechanisms of bone repair. Structural basis and biological process of bone regeneration. Linking bone healing mechanisms to biomaterial design and material selection. Created with BioRender.com.

The process of bone repair is controlled by the inherent structural integrity and well‐coordinated biological cascade of the tissue. This section summarizes the skeletal structure and common injuries to establish a background. Then, we introduce in detail the continuous cellular and molecular events that drive repair, and then study how the designed biomaterials combine with these inherent mechanisms to increase the healing outcome (Figure 2).

### 2.1 Bone Structure and Bone Injuries

The skeletal system provides critical structural support through a complex organization of bone tissue, bone marrow, neurovascular networks, and other specialized components [13]. At the tissue level, bone derives its unique mechanical properties from a composite structure: an organic matrix rich in type I collagen confers flexibility and toughness, while an inorganic mineral phase predominantly of hydroxyapatite (HAP) provides compressive strength [14, 15]. Its hierarchical architecture is exemplified by the Haversian system, the basic structural unit comprising concentric lamellae surrounding a central canal, interconnected by canaliculi [16]. Functionally, bone tissue is categorized into dense cortical bone, which resists mechanical loads, and porous trabecular bone, which is metabolically active and facilitates mineral exchange [17].

The skeletal system provides key structural support through complex tissues composed of bone tissue, bone marrow, neurovascular network and other special components [13]. At the tissue level, the unique mechanical properties of bones come from the composite structure: the organic matrix rich in type I collagen gives the bone flexibility, while the inorganic mineral phase based on HAP provides the compressive strength of the bone [14, 15]. The Haversian system is a typical representative of its hierarchy. It is a basic structural unit composed of concentric thin sheets around the central tube and connected by a small tube [16]. Functionally, bone tissue is divided into dense cortical and porous trabecular bones. The former can resist mechanical loads, while the latter is active in metabolism and promotes mineral exchange.

Cell activity that controls bone homeosis and repair is mediated by three main cell types. Osteoblasts come from mesenchymal stem cells (MSCs), which can deposit and mineralize bone matrix. Bone matrix is a collagen‐rich precursor that can mature into bones and secrete regulatory factors affecting local bone metabolism [18, 19, 20]. Osteocytes are pre‐osteoblasts embedded in the mineralized matrix, forming a huge gap‐bone tubular network, which senses mechanical stress and regulates mineral homeosis [21]. On the contrary, osteoclasts from the hematopoietic spectrum repair bones by secreting acid and protein hydrolase [22]. The balance between bone formation and angiogenesis is the basis of continuous bone reconstruction [23].

Bone integrity can be damaged by a series of diseases, which can be roughly divided into traumatic, metabolic, infectious, neoplastic, or hereditary. Fractures caused by trauma are the most common forms of acute bone injury [24]. Osteoporosis and other metabolic bone diseases lead to increased susceptibility to fractures, especially in the elderly [25]. Infections such as osteomyelitis can lead to local damage. If it is serious, it is necessary to undergo intense interventions, including amputation [26]. Primary and metastatic bone tumors destroy the normal bone structure and usually require surgical removal, resulting in substantial bone defects [27, 28]. Genetic and epigenetic abnormalities in skeletal developmental disorders can lead to structural abnormalities and damage bone function [29, 30, 31]. A common serious consequence of these different causes is bone defects that exceed the inherent regenerative ability; therefore, external intervention is needed to achieve functional recovery.

### 2.2 Bone Repair Mechanisms

Bone repair goes through three stages: inflammation, cartilage scab formation, hard bone scab formation and bone remodeling [32]. It begins with hematoma formation and local inflammatory reaction. Macrophages infiltrate the damaged site, playing the role of removing cell fragments and containing pathogens, while secreting cytokines and coordinating the subsequent regeneration cascade [33]. Macrophage polarization is considered to be a central regulatory event in the process of bone healing. In the early stage, M1‐like macrophages help to debris removal, pathogen defense and activation of inflammatory signals, and the subsequent transformation to M2‐like phenotype is crucial to inflammation subsidence, angiogenesis, MSC recruitment and osteogenic differentiation. Therefore, this timely transformation from promoting inflammation to promoting a regenerative immune microenvironment is crucial for coordinating bone repair [34, 35]. Therefore, the design of bone repair hydrogel should focus on bone formation and immune regulation, especially the regulation of macrophage behavior and phenotyping. Raggatt et al. has confirmed the key function of macrophages in coordinating this early stage and initiating tissue repair [36]. After inflammation, the repair process transitions to the cartilage scab stage. Through proliferation and cartilage matrix secretion, chondrocytes form cartilage scabs to fill and connect the fracture gap. This temporary structure, such as femoral fracture, provides the necessary initial mechanical stability by connecting displaced bone fragments, although its bearing capacity is limited [37]. The subsequent stage of hard bone scab formation involves the gradual replacement of cartilage templates by immature bones. Osteoblasts become the main cell type, depositing mineralized bone‐like matrix and converting cartilage scabs into hard bone scabs, thus restoring the mechanical integrity of the damaged site [38]. The final reshaping stage reconstructs the newly formed bone to restore its original biomechanical ability and anatomical structure. This stage is characterized by coupling activity: osteoclators absorb excessive or poorly arranged bones, and osteoblasts deposit new bones in a highly orderly manner [39]. This remodeling process is crucial for the restoration of all functions after injuries such as hip fractures, and it improves the internal structure of the bone [23]. This stage is not only cell activity, but also finely regulated by mechanical signals. As proved by Zou et al., the mechanically sensitive ion channel Piezo1 plays a key role in the fine regulation of bone homeosity by regulating the interaction between osteoblasts and osteoclators under physiological load [40].

### 2.3 Bone Repair Materials

The innate self‐healing ability of bone is often insufficient in the cases of critical‐sized defects or damaged healing. The application of bone repair materials aims to overcome these limitations by providing a supportive microenvironment that actively promotes integration with host tissue and guides the regeneration process. The material characteristics used for this role include strong biocompatibility and controllable biodegradability, which together promote stable integration, support new tissue growth, and minimize adverse immune responses [41]. The evolution of bone repair materials reflects the efforts of equilibrium mechanics and biological requirements. Metal implants, including bone screws and bone plates, have long been the standard for stabilizing load‐bearing defects in parts such as the femur and spine, mainly because of their superior mechanical strength [42]. Recent progress, taking the work of Wang et al. as an example, highlights the potential of bioactive metals; their single‐cell analysis shows that high‐purity magnesium implants enhance the repair of osteoporotic fractures by regulating the cell heterogeneity and transcription kinetics of the damaged site [43]. Despite these advances, the basic biological inertness of metal often prevents it from fully integrating with the surrounding bone tissue [44]. This restriction has promoted the development of bioactive ceramics, such as calcium phosphate (CaP), which closely imitates the mineral phase. The bone conductivity, biodegradability and inherent protein affinity of CaP make it an attractive substitute for bone grafts [45]. For example, He et al. developed amorphous CaP nanoparticles by a low‐temperature aqueous solution method, proving their dual ability to enhance osteogenesis and angiogenic activity in vitro [46]. However, the clinical application of CaP‐based materials is often limited by inherent brittleness and insufficient mechanical strength under load‐bearing scenarios. HAP is another key bioceramic. It has good biocompatibility and can be directly combined with bones [47]. Innovative manufacturing technologies, such as the incorporated HAP bioink for 3D printing developed by Ren et al., have solved the problem of material uniformity and mechanical properties of bone tissue engineering brackets [48, 49]. However, the challenges associated with low fracture toughness and precise control of macroscopic and microporosity still limit its wide application in large‐segment bone defect regeneration. Considering the limitations of single‐component systems, important research interests have shifted to composite materials and polymer‐based strategies. The hydrogel is composed of a hydrophilic polymer network, which provides a highly adjustable platform to simulate the hydration environment of natural ECM. They have good biocompatibility, cell encapsulation ability and tissue regeneration ability [50]. The advanced hydrogel design, such as the dynamic GelMA/DNA dual network system reported by Zhu et al., integrates biochemical signals and adaptive mechanical properties to guide the formation of woven bone‐like tissue [51]. This method reflects the broader trend of bone tissue engineering, that is, combining structural scaffolds, bioactive factors and stem cells to reproduce the complex bone healing microenvironment [52]. Gowsihan et al. emphasized that hydrogels are valuable in providing basic biophysical stimuli that determine the fate of cells and support tissue healing in vitro and in vivo [53]. These innovative methods make personalized treatment plans for complex defects possible, surpassing the ability of traditional repair materials.

## 3 Construction Strategies of Hydrogels for Bone Repair

**FIGURE 3:**
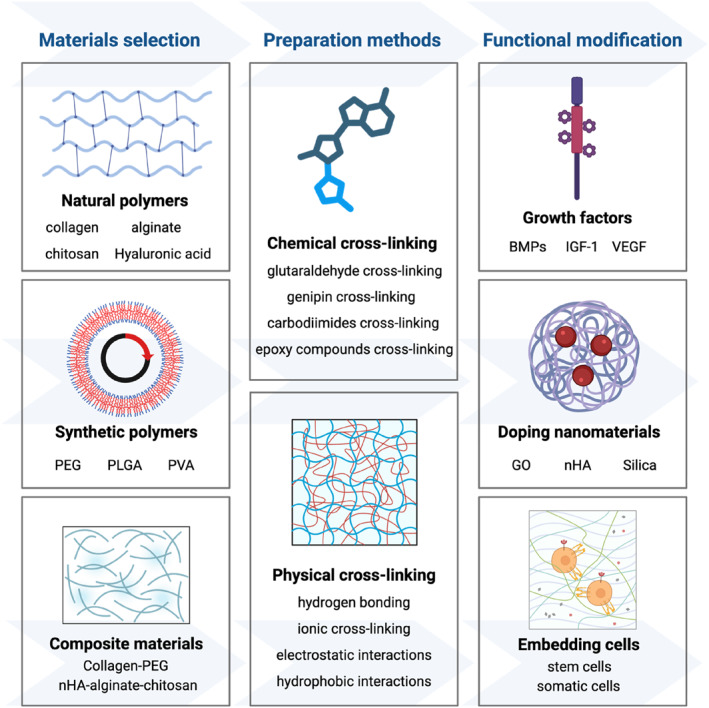
Construction strategies of hydrogels for bone repair. Integrating material selection, preparation methods, and functional modification for enhanced bone repair. Created with BioRender.com.

The advanced treatment for bone repair puts the hydrogel system at the forefront of tissue engineering research. As an adjustable system for controlled cell and biologically active molecular delivery, hydrogels are particularly effective in treating complex bone defects [54]. The ideal hydrogel for bone repair should have inherent non‐cytotoxicity and immune inertness, while providing bone conduction support, and under appropriate circumstances, the ability to induce osteogenic signals and differentiation [55]. It is crucial to summarize the key aspects of ECM to support cell behavior. The material needs to show sufficient structural integrity and mechanical strength to withstand biomechanical loads and standard sterilization procedures without compromising its function [56]. This section provides the main strategies to meet these multifaceted needs, focusing on three basic pillars: reasonable material selection, tailor‐made preparation methods, and precise functional modification (Figure 3).

### 3.1 Materials Selection

The selection of component material is the basic determinant of bone repair hydrogel design, which directly determines its final biocompatibility, structural integrity and therapeutic performance. This selection mainly includes three different categories, each of which provides a set of unique advantages. Natural polymers have inherent biocompatibility and inherent biological activity, and their biochemical similarity with bone ECM helps better cell identification, adhesion and differentiation [57]. Synthetic polymers have a high degree of chemical and physical customization. Their mechanical robustness, biodegradation dynamics and therapeutic release behavior can be precisely designed to meet the needs of different stages of the healing process, ensuring predictable performance and structural stability [58]. In order to overcome the inherent limitations of pure natural or synthetic systems, composite materials have been developed. Natural and artificially synthesized incorporated polymers combine bio‐guided characteristics, mechanical elasticity, and adjustable design to provide a more balanced and versatile regeneration path for [59]. The following section gives a strict inspection of these three material categories and explains their specific applications.

#### 3.1.1 Natural Polymers

Natural polymers constitute the main material platform for hydrogels for bone repair. Natural biocompatibility, biodegradability and structure similar to ECM are the main reasons for its wide application. This bionic property creates an ecm‐like microenvironment that supports the adhesion, growth and differentiation of osteoblasts [60]. Its key functional advantage lies in the existence of an inherent biological activity matrix [61].

##### 3.1.1.1 Collagen

Collagen is the major protein in bone ECM and is pivotal in the structure and function of bone tissue [62]. Collagen hydrogel supports the activity of osteoblasts. It can also be designed to have sufficient mechanical strength and controllable degradation characteristics [63]. In the defect model of critical size of mice, Rosa et al. proved that implanted collagen scaffolds enhanced bone regeneration [64]. To further promote the platform, Chen et al. have developed collagen CaP‐nanoparticles. They can effectively drive MSC ossification and induce ectopic ossification in vivo [65]. Even simplified synthetic analogs show prospects. García et al. reported that a cell‐free and growth factor coating based on collagen‐simulated peptides significantly promoted the repair of large bone defects, emphasizing the inherent biological activity of collagen‐derived signals [66].

##### 3.1.1.2 Chitosan

Chitosan is a positively charged polysaccharide obtained from acetylation of chitin [67]. Its positive charge is conducive to electrostatic interaction with anionic biomolecules and cell membranes, enhancing cell adhesion, and making it an effective delivery carrier of nucleic acids and growth factors [68]. The work of Li et al. proves this multifunctionality. Chitosan hydrogel containing bone morphogenetic protein‐2 (BMP‐2) can effectively promote the osteogenesis differentiation of MSCs and accelerate the healing of skull defects in rats [69]. Lelkes et al. created electrostatic spinning chitosan brackets containing HAP, which can simulate the periosteum and promote bone repair of critical size defects [70].

##### 3.1.1.3 Alginate

Alginate is a polysaccharide derived from brown seaweed, which can be easily gelled into a hydrogel by cross‐linking with mild ions of divalent ions (e.g., Ca^2+^). It makes alginat hydrogels beneficial to the encapsulation of cells and drugs [71, 72]. Its non‐immunogenic and mild processing conditions are ideal for maintaining the activity and function of capsule therapy [73]. In bone repair, alginate hydrogel forms a water‐rich porous structure, promotes the diffusion of nutrients, and acts as an effective delivery matrix [74]. Alsberg et al. have developed photointerlinked alginate derivatives with adjustable degradation and mechanical properties to support the vitality and proliferation of bone marrow mesenchymal stem cells (BMSCs) [75]. More complex systems, such as the injectable thermo‐responsive alginate hydrogel developed by Carmen et al., which is loaded with a variety of osteogenic factors, including 17β‐estradiol, BMP‐2 and plasma rich in growth factors, have shown the effect of promoting bone regeneration in osteoporosis rat models [76]. These advances highlight the utility of alginate as a multifunctional matrix, which can be combined with other biological materials to achieve better repair effects.

##### 3.1.1.4 Hyaluronic Acid (HA)

HA is a glycosaminoglycan in connective tissue, which is pivotal in tissue hydration, cell migration and wound repair [77]. HA‐based hydrogels are valued in bone tissue engineering because of their high biocompatibility, low immunogenicity and support for osteogenic differentiation [78]. Through direct modification, HA can be designed to regulate stiffness, biodegradation dynamics and release characteristics, making it an excellent carrier for therapeutic loads [79]. Zhu et al. developed graded hydrogel composites by embedding microspheres wrapped in alenphosphinate into the methyl methacrylate HA matrix. The resulting structure shows good cell compatibility and significantly promotes the osteoblastic response, indicating the strong potential to repair osteoporotic defects [80]. In the critical size rat skull defect model, Michael et al. used HA hydrogel containing inactivated tendon particles to enhance bone repair. Compared with the control group, the volume of new bones increased significantly [81]. These examples emphasize the ability of engineered hyaluronic acid hydrogels to provide complex biochemical and structural clues for bone regeneration.

#### 3.1.2 Synthetic Polymers

Synthetic polymers provide a key basis for bone repair engineering hydrogels, supporting unparalleled control in biodegradability, mechanical properties and processing technology. It is precisely defined chemical properties allow regulation of the network structure, pore structure and degradation characteristics. It can also control the diffusion of nutrients, cell‐material interaction and the continuous release of osteogenic factors. The synthetic source of these materials ensures high inter‐batch repeatability and scalability, which is a key attribute for the development of standardized and clinically convertible regenerative therapies.

##### 3.1.2.1 Polyethylene Glycol (PEG)

PEG is a highly versatile skeleton designed as a hydrogel. Its structure can be designed to adjust mechanical strength, degradation dynamics and hydrophilicity, so as to create a bracket with specific application properties [82]. The main advantage of PEG configurations is their predictable biodegradability, which contributes to temporary mechanical support during healing without the need for secondary removal surgery [83]. Wu et al. developed an injectable tetra‐PEG hydrogel, which is characterized by rapid gel formation, high strength and excellent biocompatibility. It significantly enhances the preservation and regeneration of alveolar bones in the anticoagulation rat model, highlighting its potential for oral and cranial applications [84]. Jeffrey et al. further proved the effectiveness of the PEG matrix as a template for host cell implantation and remodeling. After PEG hydrogel loaded with recombinant human BMP‐2 was implanted in the cranial defect of rats, endogenous cells were fully infiltrated and reshaped into natural bones within 5 weeks [85]. The inherent hydrophilicity of PEG also promotes a highly hydrated microenvironment conducive to cell viability and bone formation [86]. In order to promote this concept, Li et al. designed a complex PEG‐based system, which was co‐loaded with BMP‐2@ zeolitic imidazolate framework‐8 (ZIF‐8)/PEG‐NH_2_ nanoparticles and platelet‐derived growth factor‐BB (PDGF‐BB). This design uses the hydrophilicity of PEG to enhance nutrient exchange and factor stability, and realize the continuous release of factors, so as to achieve significant vascular bone regeneration in critical‐sized defects [87]. Accordingly, PEG‐based hydrogels are customizable platforms for the controlled delivery of bioactives and support of complex bone regeneration.

##### 3.1.2.2 Poly(lactic‐co‐glycolic Acid) (PLGA)

PLGA is valued for its clear degradation behavior and biocompatibility [88]. By changing the ratio of lactic acid and glycolic acid, the degradation rate of PLGA can be accurately adjusted, so as to adjust the release curve of the wrapping agent and make it highly adapted to different treatment timelines [89]. PLGA‐based hydrogels provide the necessary mechanical stability for the regenerative tissue environment [90]. For example, Ji et al. designed a bionic PLGA scaffold enhanced with white stone nanoparticles, which provides strong structural support and significantly accelerates the differentiation of osteoblasts and bone formation of rat tibia defects [91]. The controlled degradation of PLGA is strategically used for continuous drug delivery [92]. An injectable Mg@PEG‐PLGA hydrogel proved by Zhou et al. releases hydrogen and magnesium ions after degradation. It can also effectively remove reactive oxygen, regulate inflammation, and promote the regeneration of osteoporotic bone defects through a dynamically regulated osteogenic microenvironment [93].

##### 3.1.2.3 Polyvinyl Alcohol (PVA)

PVA is noted for its film‐forming ability, mechanical strength and water solubility. It can form a stable and elastic hydrogel after cross‐linking, which is attractive to bone tissue engineering [94]. The high flexibility of PVA allows it to be designed to have mechanical behavior similar to natural bones, which makes it suitable for scaffolds that require structural integrity and controlled delivery of biological agents. This adaptability has been proved in a hybrid network hydrogel composed of PVA, PLGA, chitosan and sodium alginate. Its Young's modulus is 47.5 ± 5 kPa, which significantly enhances the scaffold stiffness, thus promoting osteogenic differentiation [95]. Nagarajan et al. further realized the functionalization. They developed a PVA‐based hydrogel loaded with chitosan nanoparticles containing quinopyl. The system provides continuous release of qinpixin, activates the Wnt/β‐catenin signaling pathway to promote in vitro osteogenesis, and stimulates a large number of new bone formation in the rat tibial defect model [96].

#### 3.1.3 Composite Materials

By combining the biological activity and ECM simulation properties of natural polymers with the mechanical elasticity and design flexibility of synthetic polymers, the composite hydrogel achieves superior overall performance in terms of mechanical strength, biological function and controlled degradation [97]. For example, the combination of collagen and PEG produces a composite material that shows stronger mechanical strength without sacrificing the basic biological clues provided by collagen for cell adhesion and differentiation. PEG components help to be hydrophilic and allow fine‐tuning mechanical properties through changes in molecular weight and cross‐linking density. Qian et al. developed a three‐component bionic hydrogel containing PEG‐Polycaprolactone (PCL)‐PEG copolymer, collagen and nanohydroxyapatite (nHA). Implanting this composite scaffold into rabbit skull defects for 20 weeks shows that compared with simple natural repair, this composite scaffold significantly enhances bone regeneration and guides bone healing [98]. Incorporating nHA into natural polymer hydrogels such as alginate or chitosan is a widely used strategy to improve bone conductivity and mechanical properties. nHA particles can not only enhance the structure of the scaffold, but also enhance their biocompatibility by promoting osteoblast differentiation and matrix mineralization. Zhang et al. developed an nHA‐chitosan complex hydrogel, which significantly improved osteoblastic activity, cell adhesion and mineralization, and promoted bone regeneration in rabbit tibial models [99]. Further supporting this method is that a gelatin/alginate compound hydrogel loaded with nHA has a biologically active porous structure, which can promote the osteoblastic interaction between macrophages and BMSCs, thus significantly improving the regeneration of maxillofacial critical size defects and providing key supporting structures [100]. With the continuous development of materials science, the combination of natural polymers, synthetic polymers and non‐component components is expected to become more and more complex and diversified, thus providing tailor‐made treatment options for bone defects of different complexities and anatomical positions.

### 3.2 Hydrogel Preparation Methods

The method used in the preparation of hydrogel determines the ultimate physicochemical and biological properties, including the microstructure, mechanical durability, degradation dynamics and overall function. The selection between chemical crosslinking and physical crosslinking strategies mainly depends on the specific requirements of the target bone repair application [101]. Chemical cross‐linking usually produces networks with superior stability and mechanical strength, while physical cross‐linking usually provides stronger biocompatibility and inherent stimulus responsiveness, making it suitable for different regeneration scenarios [102].

#### 3.2.1 Chemical Cross‐Linking

Chemical cross‐linking establishes permanent covalent bonds between polymer chains to form a 3D network known for its high mechanical integrity and structural stability [103]. The hydrogel prepared by chemical cross‐linking has high tensile strength, lower degradation rate and stronger anti‐physiological stress ability. It is ideal for load‐bearing bone repair. Glutaraldehyde is a historically famous chemical cross‐linking agent. It reacts effectively with free amine groups on polymers (such as chitosan, gelatin and collagen) to form a stable Schiff base linkage [104, 105]. This reaction significantly improves the stiffness and compressive strength of the scaffolds. Chitosan/gellatin scaffolds cross‐linked with glutaraldehyde show better mechanical properties and effectively support the adhesion and proliferation of osteoblasts. For example, Massimo et al. confirmed that this cross‐linking significantly enhances the structural stability, porosity, swelling behavior and anti‐degradability of these supports, making them more suitable for bone repair applications [106]. However, the high cytotoxicity of glutaraldehyde requires strict post‐treatment to remove residual compounds, which is an important disadvantage that limits its clinical transformation [101]. In order to solve this toxic problem, the natural cross‐linking agent from gardenia Ellis has become a good alternative [107] Genipin also reacts with beramine, but forms a biocompatible blue network. Compared with glutaraldehyde cross‐linked hydrogel, genipin cross‐linked hydrogel has lower cytotoxicity, better biocompatibility and more favorable degradation properties. Research shows that genipin flat cross‐linking can significantly enhance the compressive strength of chitosan/collagen scaffold. It has optimal performance at a specific concentration and temperature, and the mechanical properties continue to improve with the extension of the cross‐linking time [108]. Other biocompatible chemical cross‐linking strategies have also been developed: Carbodiimides (e.g., Ethyldimethylaminopropyl Carbodiimide/*N*‐Hydroxysuccinimide, EDC/NHS): these compounds activate carboxyl groups on polymers to form amide bonds with amines under mild water conditions. This method avoids the inclusion of toxic cross‐linking molecules into the network. EDC/NHS cross‐linked collagen hydrogel is particularly attractive for bone tissue engineering because of its high biocompatibility [109]. Epoxy compounds (e.g., poly(ethylene glycol) (N) diglycidyl ether, PEGDE): PEGDE reacts with hydroxyl groups or amino groups to form a flexible and highly hydrophilic network. PEGDE‐overlinked hydrogel has been successfully used in cell encapsulation, while maintaining structural stability under physiological conditions [110].

The cross‐linking strategy of bone repair hydrogel should be evaluated by its ability to increase rigidity and structural integrity, cytotoxic characteristics, residual reactant load, degradation product safety, and conversion feasibility [111]. Among the commonly used chemical crosslinking agents, glutaraldehyde is still very effective in enhancing collagen and gelatinic matrix, but its translational attraction is limited by the recognized cytotoxicity and the need to control or neutralization of unreactive aldehyde residues [112]. Genipin provides a balance between stability and biocompatibility. But it should be described as low toxicity rather than essentially non‐toxic, because higher genipin concentrations will still reduce cell viability [112, 113]. EDC/NHS‐based cross‐linking is usually attractive for matrix stability, but it is not always superior in practice. Its performance depends on the preparation and must be judged according to the required healing time window. In a recent representative comparison, edc cross‐linked gelatin hydrogel has a faster gelatinization rate, but it degrades within 24 h, while the optimized genipin cross‐linked hydrogel remains structurally available after 7 days and supports > 70% of cell viability [114]. It is important that the safety discussion should go beyond acute in vitro toxicity. The cross‐linking density itself can reshape the host reaction in vivo. Compared with mildly cross‐linked matrices, severely cross‐linked hydrogels induce greater inflammation, increased macrophage fusion, and enhanced fibroblast activity [115]. Degradation by‐products should be regarded as biologically active variables, not inert waste, because recent evidence shows that acidic hydrogel fragments can change the survival and activation status of macrophages [50].

These studies show that it is a feasible application to preserve glutaraldehyde for maximum fixation and detoxification after the reaction. Genipin is usually a more practical compromise solution that requires long‐term stability of natural polymer bone repair hydrogel, and EDC/NHS should only confirm sufficient persistence and biology in formula specificity data. The selection of cross‐linking agents should be defined as translation‐oriented design decision‐making of balanced reinforcement, residual risk control, host compatibility and degradation behavior, rather than purely mechanical optimization steps.

#### 3.2.2 Physical Cross‐Linking

Physical cross‐linking is a key strategy for constructing a hydrogel network through reversible non‐covalent interactions (including hydrogen bonding, ionic colocation, electrostatic and hydrophobic bonding) [101]. This method avoids the use of cross‐linking reagents that may be cytotoxic and is carried out under mild physiological conditions. The inherent reversibility of these physical bonds gives the hydrogel dynamic and stimulus response characteristics, such as sensitivity to temperature, pH or ion strength. Therefore, physical cross‐linked hydrogel is particularly suitable for wrapping living cells, growth factors and other unstable biologically active molecules, so as to maintain their activity and functionality [116]. A typical example is the formation of alginate hydrogel through ion cross‐linking. When the sodium alginate solution encounters divalent cations, such as calcium ions (e.g., calcium chloride), the cations bridge the carboxylic acid groups on the adjacent sodium alginate chain to induce rapid sol‐gel transformation [117]. This mild gelation process helps to effectively encapsulate and the subsequent controlled release of osteogenic factors or stem cells. The dynamic properties of ion cross‐linking can be adjusted or reversed by chelating agent. They provide a method for fine‐tuning the properties and degradation curves of hydrogels [118]. Thermo‐responsive hydrogel represents another important physical cross‐linking system. Po Polymers like poly(N‐isopropylacrylamide) (PNIPAAm) exhibit reversible sol‐gel transformation determined by temperature. At its lower critical solution temperature (LCST, about 32°C), the PNIPAAm chain was hydrated and soluble. When heated above LCST, due to the enhancement of hydrophobic interaction, the chain is dehydrated and collapsed, resulting in physical gelation [119]. This characteristic makes it possible to prepare injectable hydrogel, which is liquid at ambient temperature and easy to operate and inject, but can form a stable gel in situ under body temperature, providing an important prospect for minimally invasive bone repair surgery.

Integrating physical and chemical cross‐linking mechanisms into a single matrix, usually through a network architecture that penetrates each other. It represents an advanced method that overcomes the limitations of using any method alone. This collaborative design can achieve strong mechanical stability (provided by covalent networks) and dynamic, cell‐led remodeling (provided by reversible physical interactions) at the same time [120]. A primary covalent crosslinking network establishes permanent structural integrity and strength to withstand the physiological load in this hybrid system. The secondary physical cross‐linking network introduces dynamic characteristics to enable adaptive reshaping. It also stimulates responsive release of biologically active goods, and interaction with invading cells [121].

### 3.3 Functional Modification of Hydrogels

Targeted functional modification enhanced the therapeutic effect of hydrogels for bone repair. Integrating bioactive molecules, nanomaterials and embedding living cells are the three basic strategies to improve the function of hydrogel. This modification enables researchers to precisely adjust the properties of hydrogel to solve specific challenges in bone regeneration, including limited bone induction ability, insufficient mechanical strength and poor integration with host tissue.

#### 3.3.1 Growth Factors

Growth factor is the signaling protein that regulates behaviors such as cell proliferation, differentiation and matrix synthesis in the process of bone regeneration. Incorporating these growth factors into a hydrogel can enhance its ability to support bone healing and improve osteogenesis and angiogenesis activity in bone repair [122].

##### 3.3.1.1 Bone Morphogenetic Proteins (BMPs)

BMPs, especially BMP‐2 and BMP‐7, have bone‐induced characteristics, which can promote the differentiation of mesenchymal stem cells into osteoblasts [123, 124]. When BMP‐2 is wrapped in gelatin, it has been proven to provide continuous release, promote osteoblast differentiation and enhance bone regeneration of bone defects of critical size [125]. A large number of studies have confirmed that BMP‐2 is crucial to the bone repair function modification of hydrogel, and is a key signal to activate the repair response of host and plant periosteal cells. In its absence, callus formation is impaired. Increasing the availability of local BMP‐2 can promote new bone formation and minimize the fibrosis of the host‐plant interface, thus significantly improving the results of plant integration and healing [126, 127, 128]. BMP‐7 has been found to be particularly effective in cartilage repair and bone mineralization enhancement in damaged bone areas [129, 130]. In animal models, chitosan hydrogel loaded with BMP‐7 improves bone healing by stimulating osteoblast proliferation and promoting bone matrix deposition. BMP‐7 has been widely used in bone repair hydrogel construction as a bone inducer, and clinically used in long bone fracture non‐healing, spinal fusion and oral maxillofacial surgery. When combined with hydrogel or other tissue engineering scaffolds, BMP‐7 provides continuous delivery and promotes bone formation [124, 131].

##### 3.3.1.2 Vascular Endothelial Growth Factor (VEGF)

VEGF is mainly used as the regulating factor of angiogenesis, establishing the necessary vascular network to supply oxygen and nutrients to the regenerated bone tissue. Incorporating it into alginate hydrogel can effectively promote the formation of new blood vessels at the injured site, so as to accelerate the bone healing process by improving the nutritional supply [132]. In addition to the effect of angiogenesis, there is evidence that VEGF directly affects the function of osteoblasts by promoting proliferation, collagen production and mineral deposition in the process of osteoblast differentiation [133]. This dual function makes VEGF a key regulating factor for bone repair in hydrogel, which can support vascularization and osteogenesis at the same time. However, to achieve the best therapeutic effect, it is necessary to precisely control the release kinetics of VEGF, because uncontrolled release may lead to abnormal mineralization patterns and damage to bone mass [134].

##### 3.3.1.3 Insulin‐like Growth Factor‐1 (IGF‐1)

IGF‐1 accelerates de novo bone formation by synergistically stimulating the proliferation and differentiation of osteoblasts and plays a key role in bone regeneration [135]. In order to maintain its biological activity and achieve local delivery, IGF‐1 has been successfully integrated into the PLGA hydrogel system, providing an ideal controlled release for bone repair applications. Compared with the control group, the defects of implanting the critical size of the PLGA hydrogel experiment loaded with IGF‐1 significantly enhanced the proliferation of osteoblasts, alkaline phosphatase activity and mineralized matrix deposition [136]. The sustained release properties of the PLGA matrix prolong the biological activity of IGF‐1 and ensure continuous osteogenic stimulation throughout the repair process. The advanced delivery system has further improved this method. As proved by Yin et al., double‐layer PLGA/chitosan scaffolds release BMP‐2 and IGF‐1 in an orderly manner in the early healing stage, followed by continuous release of BMP‐2 [137]. This time control guides human adipose‐derived stem cells to form coordinated osteochondral tissue through different stages of cartilage and osteogetic differentiation.

BMP‐2, BMP‐7, VEGF and IGF‐1 are often discussed as pro‐regenerative cues. Their bioactivity should be explained within a dose‐dependent safety framework and not a single benefit. Recombinant human BMP‐2 and BMP‐7 are one of the few bone‐induced proteins that have reached clinical applications. However, recent studies and reviews emphasize that the therapeutic effect often depends on superphysiological doses, especially when collagen‐based carriers exhibit burst release, which may lead to ectopic ossification, edema and inflammation, bone resorption, local compression, and a substantial cost burden [138, 139]. These transformational limitations are not just theoretical: Osigraft based on BMP‐7 was eventually terminated, even though it had been clinically adopted before, which emphasized that regulatory approval does not necessarily guarantee lasting clinical success [140]. The treatment window of VEGF is also very narrow. In engineered bone, only a strictly controlled dose of VEGF combined with the matrix can promote the coordination of vascular invasion and osteogenic differentiation, while higher doses will increase the collection of osteoclasts and inhibit osteogenic differentiation, indicating that excessive VEGF will separate angiogenesis from bone formation, rather than promote bone formation [141]. Additionally, the dysregulated VEGF signaling is associated with pathological angiogenesis and increased vascular permeability, which highlights the risk of uncontrolled exposure even under angiogenesis stimulation aimed at promoting repair [142]. The direct clinical transformation of IGF‐1 in bone repair is still limited; this caution is biologically reasonable, because recent evidence shows that local IGF1 over‐activation can drive wnt‐dependent joint damage and abnormal hypertrophy reactions, indicating that when space and time control is lost, anabolic signals may become pathological [143]. Therefore, the discussion on the functionalization of growth factors should balance the regenerative efficacy and safety, manufacturability and regulation, and should emphasize local delivery, burst‐release control and factor‐specific treatment windows, rather than a simple dose increase.

#### 3.3.2 Doping With Nanomaterials

The strategic combination of nanomaterials and hydrogel matrix enhances the scaffold function for bone regeneration. Doping of nanomaterials significantly improves mechanical properties, biological activity and bone conductivity through nano‐level interaction with polymer networks and biological environments [144]. The integration of graphene oxide (GO) in hydrogel provides special mechanical reinforcement and enhances biological activity. The two‐dimensional structure and surface chemistry of GO are conducive to the strong interface interaction with the polymer chain, which greatly improves the compressive strength and elastic modulus. The oxygen‐containing function group of GO promotes ossification and differentiation through a variety of signaling pathways. Gelatin/GO composite hydrogel showed this dual function, which significantly enhanced osteoblast proliferation and differentiation markers, and finally accelerated bone regeneration in the preclinical model [145]. The incorporation of nHA creates a bionic composite material, which closely replicates the natural bone mineral environment. The similarity between nHA and bioapatite gives them special bone conduction properties, which directly promotes mineralization and osteoblast adhesion. Alginate/nHA composite is an example of this method, which significantly enhances bone formation in the body by improving cell‐mate interaction and mineral deposition [100]. nHA incorporated with PCL hydrogel provided a biologically active surface and promoted the formation of bone tissue in critical size defects. Wang et al. revealed that PCL/nHA composites not only promote the osteogenic differentiation of BMSCs, but also induce the beneficial polarization of macrophages to the M2 phenotype, thus establishing a regenerative immune microenvironment that can enhance bone integration in the skull defect model [146]. Silicon nanoparticles contribute to the mechanical enhancement and biological enhancement of the hydrogel system. Their integration increases the compression modulus and creates favorable surface characteristics for cell adhesion and differentiation. In bone regeneration research, PEG hydrogel doped with silicon showed an improvement in bone formation. Advanced applications include multi‐functional systems, such as PEG hydrogel containing mesoporous silicon dioxide nanoparticles developed by Song et al. for diabetic periodontal regeneration. This complex design uses the continuous release of stromal cell‐derived factor‐1 to recruit endogenous BMSCs, while releasing metformin to remove reactive oxygen and combat the osteogenic effect of diabetes damage [147, 148]. Similarly, chitosan/silica nanocomposite hydrogel creates a microenvironment that significantly enhances the activity and bone repair ability of osteoblasts by combining morphology and biochemical characteristics.

#### 3.3.3 Embedding Cells

Embedding cells into hydrogels can provide a direct source of osteoblasts at the injured site, significantly enhancing biological integration and bone regeneratio [149]. This method can be roughly divided into two strategies: implanting stem cells and somatic cells.

##### 3.3.3.1 Stem Cells

MSCs are multipotent cells with high bone‐generating potential. MSCs loaded in collagen hydrogel have successfully regenerated the critical‐sized mandible defects of pigs, demonstrating their potential in large animal models [150]. Takashi et al. found in the rat femoral defect model that mesenchymal stem cells wrapped in alginate hydrogel can not only promote bone formation, but also stimulate angiogenesis, thus promoting vascularization and bone regeneration at the same time, thus improving the overall healing environment [151]. Induced pluripotent stem cells (iPSCs) can be differentiated into a variety of cell types, providing an alternative for the application of adult stem cells. iPSCs‐derived osteoblasts embedded in PEG hydrogel have been shown to repair cranial defects in mice and produce a large number of new bone volumes. Abdullah et al. developed a human bone marrow organoid model derived from iPSCs, encapsulated in hydrogels, which successfully mimicked the hematopoietic environment and supported the growth of multiple blood cell lineages and vascular structures, providing a robust system for modeling both healthy and pathological bone marrow conditions, including bone repair and hematopoiesis [152, 153]. Adipose‐derived stem cells (ADSCs) can be easily isolated from the patient's adipose tissue. ADSCs implanted with PVA hydrogel showed enhanced osteogenic and collagen synthesis in rat skull defects, providing a less invasive alternative method for other types of stem cells, while maintaining significant regenerative potential. Lakshmi et al. proved that the amniotic hydrogel loaded with ADSCs effectively reduced inflammation and cartilage degeneration in the rat model of osteoarthritis, and promoted cartilage regeneration by combining the anti‐inflammatory and cartilage protection properties of adsc and amniotic hydrogel, highlighting its potential for bone repair [154, 155].

##### 3.3.3.2 Somatic Cells

Osteoblasts are responsible for bone formation. Due to their direct effect on bone regeneration, they are often used in hydrogels. The incorporation of osteoblasts into gelatin hydrogel has been proven to accelerate the mineralization and defect repair of critical cranial defects [156]. In addition, the chitosan hydrogel loaded with osteoblasts is rapidly integrated with the host bone, indicating that the embedded osteoblasts can not only enhance bone matrix deposition, but also improve bone integration at the defect site [157]. Although fibroblasts are not direct osteoblasts, they play a key role in tissue repair by promoting ECM remodeling. Studies show that chitosan hydrogel loaded with fibroblasts can improve wound healing and soft tissue regeneration around bone defects, indirectly providing a more favorable environment for the function of osteoblasts [158]. Chondrocytes can be embedded into hydrogels to promote cartilage regeneration for osteochondral defects. Chondrocyte‐laden hydrogels composed of collagen and alginate have been shown to enhance cartilage formation in osteochondral defect models, providing a dual benefit by supporting both cartilage and bone regeneration in complex joint defects [159]. The embedding of either stem cells/somatic cells into hydrogels offers unique advantages for bone regeneration. Stem cells provide an adaptable, multipotent source for osteogenesis and can be combined with specific hydrogel properties to enhance both bone tissue repair. Somatic cells can be used for more targeted regeneration, directly contributing to bone mineralization and tissue integration.

## 4 Characterization of Hydrogels for Bone Repair

**FIGURE 4:**
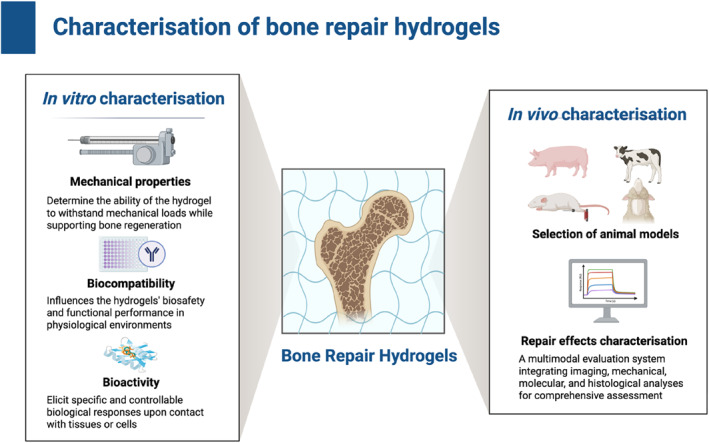
Characterization of hydrogels for bone repair. Integrating in vitro and in vivo evaluations for mechanical, biological, and functional performance. Created with BioRender.com.

In bone repair, hydrogel‐based therapy ranges from laboratory research to clinical application. A strict and multi‐faceted characteristic framework is indispensable. This process requires a comprehensive evaluation of mechanical properties, biocompatibility and regeneration potential to establish a clear association between material design and functional properties [160]. Establish such a robust evaluation paradigm to ensure that the candidate hydrogel meets the strict requirements of the physiological environment. Therefore, the combination of carefully designed in vitro analysis and biologically related in vivo models forms the cornerstone of this evaluation process (Figure 4).

### 4.1 In Vitro Characterization

In vitro identification is a basic screening stage that evaluates hydrogel under specific laboratory conditions to determine its inherent mechanical properties, biocompatibility and biological activity, which is isolated from the complexity of living organisms [161]. This controllable method provides key initial data on the properties of hydrogels in a simplified biological context, which lays the necessary premise for subsequent in vivo research that consumes more resources.

#### 4.1.1 Mechanical Properties

The mechanical properties of hydrogel directly determines the structural support throughout the repair process [162]. Key parameters include compressive strength, elastic modulus, and viscoelastic behavior. They must be designed to be close to the mechanical contours of natural bone tissue [163]. The compressive strength defines the resistance of the hydrogel to deformation under axial load, which is an indispensable feature to maintain the integrity of the load‐bearing bone [164]. The elastic modulus, as a quantitative measure of hardness, further determines the level of mechanical support provided to the healing bone. The swelling behavior of hydrogel profoundly changes its mechanical properties. Water absorption causes network expansion, which in turn reduces the effective cross‐linking density and changes the rigidity and internal structure [165]. This expansion phenomenon also affects the spatial distribution of encapsulated bioactivators and can aggravate the mechanical damage in the dynamic loading state [166]. The relevant increase in mesh size controls basic biological processes such as cell migration and tissue integration [167]. Therefore, the comprehensive mechanical evaluation needs to evaluate the swelling rate and grid topology at the same time to accurately predict the in‐body performance. Rheological analysis further reveals that the viscoelastic characteristics of hydrogels that change over time [168]. Shear rheometers and other technologies quantify the response of materials to deformation, including the energy dissipation characteristics as a key indicator of mechanical stability under cyclic stress [169]. This characteristic is especially suitable for applications involving dynamic loading defects. In this case, the hydrogel must withstand the physiological force of fluctuations without structural failure. From the perspective of transformation, the target mechanical window of the bone repair hydrogel should be determined according to the anatomical site and load‐bearing requirements. The apparent elastic modulus of the trabecular is about 10–3000 MPa, and the compressive strength is about 0.1–30 MPa, while the hardness and strength of the bone cortex are significantly higher, the elastic modulus is about 5–15 GPa, and the compressive strength is about 100–150 MPa. In contrast, most traditional hydrogels remain in the range of kPa to low mPa, which shows that their mechanical properties are generally not enough to be directly used for load‐bearing cortical repair. Even the enhanced hydrogel system can usually only approach a lower range of bone trabecular, unless it is further enhanced by mineralization, composite reinforcement or double network design. Therefore, the mechanical applicability of the hydrogel should be evaluated in a specific defect and application‐oriented manner, rather than a general standard for a single “bone‐mimetic” performance.

Beyond providing structural support, hydrogel mechanical parameters also exert instructive effects on cell fate and vascularization. Rather than treating crosslink density, modulus, and degradation as independent descriptors, recent hydrogel studies suggest that these parameters converge on a common traction‐remodeling‐stability axis. Increasing effective crosslink density can enhance local force transmission, but excessive or poorly exchangeable crosslinking may suppress cell‐driven network reorganization and restrict macromolecular diffusion. Accordingly, hydrogels with fast‐dissociating dynamic crosslinks support rapid cell spreading within 18 h and enhance mechanosensing‐dependent osteogenesis, whereas spatially heterogeneous designs that combine locally stiff anchorage with a more permissive surrounding network appear more effective than uniformly stiff matrices [170]. For example, shell‐hardened macroporous hydrogels exhibited local shell moduli of ∼28.7–163.2 kPa while the surrounding matrices remained at ∼12.3–63.3 kPa, and the shell lost only ∼20% of its modulus over 42 days compared with > 70% softening of the matrix, enabling sustained osteogenic signaling even under low bulk stiffness [171]. Likewise, MMP‐degradable microporous PEG hydrogels with G′ values in the ∼30–421 Pa range and 5–20 μm pores promoted 3D network formation within 24 h, higher ALP and RUNX2 expression, more mature collagen deposition, and greater mineralization than non‐degradable controls, indicating that degradation must be permissive enough for cell‐mediated remodeling but not so rapid that mechanical guidance collapses [172]. For angiogenesis, this relationship is also non‐monotonous: endothelial growth depends not only on hardness, but also on matrix plasticity. In collagen‐HA hydrogels with matching initial stiffness, the medium‐plastic matrix produces the longest invasion distance and the largest lumen, while the highly plastic matrix over‐activates the integrin‐focal adhesion kinase (FAK)‐contractility signaling and destroys β‐catenin/VE‐cadherin connection, and reduces the stability of blood vessels [173]. These findings support the definition of quantitative design windows for balancing local stiffness, network reorganization and time structure persistence, rather than assuming that simply increasing stiffness or slowing down degradation will uniformly improve the osteogenic‐angiogenic coupling.

#### 4.1.2 Biocompatibility

Biocompatibility requires that hydrogels remain functional in the physiological environment [174]. The standard evaluation begins with the cytotoxicity test. This determines whether the hydrogel or its degradation products have a harmful effect on the surrounding cells or tissues. Cell proliferation analysis is carried out to check the ability of materials to support cell growth and maintain normal metabolic activity [175]. In vitro assessment helps to clarify whether the material triggers an inflammatory reaction, whether it binds effectively to local tissues, and whether its degradation rate meets biological demands. Immune response analysis is particularly important, because excessive inflammation will interfere with tissue repair. The degradation tests confirm that the material decomposes at a rate that does not compromise biosafety. Alireza et al. have developed an injectable, photocrosslinkable alginate adhesive hydrogel with adjustable mechanical properties and was used to deliver MSCs for craniofacial bone regeneration. After being implanted under the skin of mice, the hydrogel only causes a slight immune response, showing good tissue integration and controllable degradation characteristics [176]. In the rat peri‐implantitis model characterized by bone loss, the hydrogel loaded with gingival MSCs can promote almost complete bone regeneration around the implant. The combination of HAP particles and mesenchymal stem cell aggregates makes it have good biocompatibility, which not only supports cell activity and osteogenic activity, but also does not cause obvious inflammatory reactions. This feature makes the hydrogel suitable for use in the oral environment [176].

#### 4.1.3 Bioactivity

Biological activity is an inherent characteristic of hydrogel, which allows it to trigger specific and controllable biological reactions after contact with tissues or cells. This characteristic plays a key role in promoting functional recovery and supporting tissue regeneration [177]. The biological activity of hydrogels designed for bone repair is usually reflected in the two main levels of the regeneration process. The first level is bone conduction, which refers to the ability of the material as a structural scaffold to support the growth of osteocytes and blood vessels, and also guide the orderly formation of new bone tissue on its surface and inside the structure [178]. The second level is osteoinductivity, which describes the ability of materials to stimulate undifferentiated stem cells or progenital cells to differentiate into the osteogenic spectrum and form osteogenic cells [179]. In vitro characterization of biological activity usually follows the progress from basic compatibility assessment to the evaluation of specialized functions. Preliminary confirmation of material safety involves cell compatibility and toxicity analysis. Live/dead staining is often used to observe the survival state of cells. Cell Counting Kit‐8 (CCK‐)8, MTT/XTT, Alamar Blue and other methods quantitatively measure the vitality and proliferation ability of cells by detecting the metabolic activity of cells [180, 181]. In order to observe the adhesion and spread of cells on the surface of hydrogel, a scanning electron microscope is used to obtain high‐resolution images of cell adhesion, morphology and pseudofoot extension. Cytoskeletal staining under laser scanning confocal microscopy further reveals the shape of cells and the mechanical stress experienced by cells [182]. The evaluation of osteogenic differentiation ability includes early and late markers. Usually, the activity of alkaline phosphatase (ALP) is checked first to provide the initial indication for bone formation induction. The formation of later mineralized nodules can be evaluated by the evaluation of the calcification capacity by staining, and qualitative and quantitative analysis of the calcification capacity can be carried out. Molecular‐level research detects mRNA expression of ossification‐related genes using reverse transcription‐quantitative polymerase chain reaction (RT‐qPCR) and verifies protein expression and localization through Western blot and immunofluorescence staining [183, 184].

### 4.2 In Vivo Characterization

In vivo characterization is crucial to evaluate the biological properties and transformation potential of hydrogels used for bone repair. By using appropriate animal models and systematic evaluation methods, it can help to determine whether hydrogel can integrate with the host tissue and promote effective bone regeneration.

#### 4.2.1 Selection of Animal Models

The selection of a suitable animal model requires consideration of the type of bone defects, the anatomy and physiological characteristics of species specificity, clinical relevance, and ethical practicality [59, 185]. Small rodents are often used in preliminary research because of their cost‐effectiveness, fast reproduction and clear genetic background. These features make it particularly suitable for calvarial defect models that are often used to detect the bone regeneration potential and biocompatibility of hydrogel [186, 187].

Rabbit models offer advantages such as moderate body size, relatively stable bone metabolism, and ease of surgical manipulation. These characteristics allow them to be used in studies of bone and cartilage defect repair, especially in situations where more complex mechanical environments or subchondral bone reconstruction needs to be simulated [187, 188]. For example, Zhang and colleagues employed a rabbit critical‐sized trochlear defect model to evaluate the 3D‐printed biphasic PEG/β‐TCP scaffolds designed for osteochondral repair [189].

Large animal models, including goats and miniature pigs, have skeletal structures that closely resemble those of humans. They share similar proportions of cortical and trabecular bone, as well as comparable load‐bearing behaviors. These similarities make them suitable for long‐term evaluation of hydrogels in critical‐sized defects located in areas such as the femur or mandible [190, 191, 192]. Chiang et al. used a porcine femoral condyle osteochondral defect model of critical size to examine the effectiveness of gelatin microbead and collagen gel systems carrying autologous chondrocytes for cartilage repair [193]. However, the high cost and lengthy duration of experiments with large animals restrict their use to advanced preclinical validation.

Disease models established by chemical or surgical induction can simulate metabolic disorders, immune dysfunction or other disease‐related environments, so that researchers can study regeneration results and immunomodulation effects at the same time [194]. The diabetes model replicates bone defects under chronic hyperglycemia, in which hydrogels may promote bone generation and angiogenesis by regulating macrophage polarization and inflammatory signals [195]. On the contrary, the glucocorticoid‐induced osteonecrosis model simulates osteocyte apoptosis and osteogenic inhibition, making it suitable for studying how hydrogels support bone regeneration and angiogenesis [196].

Clarifying the size, geometry and implantation strategy of the defect can ensure the repeatability of the experiment and maintain the correlation of transformation. Non‐weight‐bearing areas such as skulls are suitable for early assessment of osteosis potential. The load‐bearing areas such as the femoral condyle more realistically reflect the influence of mechanics on the bone healing process [197, 198, 199]. The standardization of variables such as animal age, gender and housing conditions further reduces the variability of the experiment and increases the reliability of the results.

#### 4.2.2 Repair Effects Characterization

The repair effect should be evaluated through a multimodal and quantitative system that integrates structure, function and molecular indicators. Imaging evaluation is usually the core of the system. Micro‐computer tomography can quantitatively calculate parameters such as bone volume fraction, bone tebura thickness, bone tequine number and connection density, which together provide an accurate reflection of the degree of new bone formation and defect closure [200]. Based on these images, the 3D reconstruction further visualizes the spatial pattern and progress of bone restoration over time.

Histological and immunohistochemical analysis provide cellular and molecular supplementary information. Tissue integration, collagen deposition and neovascular formation in the defect area were observed by hematoxylin‐eosin (H&E) and Masson's trichrome staining [201]. Toluidine blue or safranin O staining is used to evaluate cartilage matrix regeneration, which is especially important in the osteochondral defect models [202]. The expression and localization of key proteins involved in osteogenic differentiation, angiogenesis and inflammatory regulation were determined by immunofluorescence and immunohistochemical target markers BMP‐2, osteocalcin and CD31 [201, 203].

The mechanical test provided direct evidence of functional recovery. Using the biomechanical test system to measure the compression modulus, yield strength and related parameters helps to determine how close the mechanical properties of the repair tissue are to those of natural bones, thus linking structural repair with functional recovery [204, 205]. Biochemical analysis, including the determination of ARS activity assays, the quantitative determination of calcium nodule formation and the determination of inflammatory cytokines based on ELISA, are used to characterize changes in osteogenic activity, metabolic state and immune microenvironment [206, 207].

For complex defects (e.g., osteochondral lesions), the macroscopic scoring system should be included in the evaluation protocol. The scoring system of the International Cartilage Repair Society is often used to grade the overall repair quality, which can distinguish between serious abnormalities, abnormalities, close to normal and normal appearance of the repair area [208]. The system has been widely used in large animal models and preclinical research, and has a good correlation with histological results, thus providing a standardized and quantitative basis for the evaluation of osteochondral regeneration materials [209, 210]. Emerging technologies have further enhanced the depth of in vivo characterization. Fluorescent labeling and tracking methods, such as calcein double labeling, allow dynamic monitoring of bone mineralization rate within a specified time interval [211]. High‐throughput methods, including transcriptome sequencing, can be used to clarify the molecular pathways and regulatory networks of hydrogel‐mediated bone regeneration [211, 212]. All experimental data require strict statistical analysis and, where appropriate, use time series analysis to capture the temporal dynamics of tissue repair. Table 1 further summarizes the representative bone repair hydrogels to facilitate the cross‐study and comparison of material types, cross‐linking strategies, mechanical properties, degradation behaviors, in vivo models and regeneration results.

**TABLE 1:** Comparative summary of representative hydrogels for bone repair.

Material type	Cross‐linking strategy	Mechanical performance	Degradation/release behavior	In vivo model	Regenerative outcome	Reference
Semisynthetic HA‐based hydrogel	Dynamic boronate‐diol cross‐linking	Shear‐thinning; self‐curing; high viscoelasticity	30 days degradation	Rat femoral defect (2 × 2 × 2 mm)	Enhanced osteogenesis and mineralization	[213]
Synthetic thermo‐responsive hydrogel	Thermo‐induced sol‐gel transition	Thermogelation at ∼22.1°C	7 days sustained release	Rabbit radial critical‐size defect	Improved osteogenesis and defect repair	[214]
Composite hydrogel	Photo‐crosslinked hybrid network	High load‐bearing capacity; G’ > *G*”	/	Rat calvarial critical‐size defect (4 mm)	Increased BV/TV and osteogenic matrix formation	[215]
Natural composite immunomodulatory hydrogel	Tetrazine‐norbornene cycloaddition	Injectable; rapid gelation (∼5 min)	/	Rat calvarial critical‐size defect (5 mm)	M2 polarization, angiogenesis, and defect healing	[216]
Inorganic‐organic multifunctional composite hydrogel	HRP/H_2_O_2_‐mediated gelation	/	Sustained ion/VEGF release	Rat calvarial defect	Enhanced osteogenesis and angiogenesis with reduced osteoclast activity	[217]
Smart‐responsive synthetic hydrogel (enzyme‐responsive)	Covalent MMP‐cleavable network	Viscoelastic network; G’ > *G*”	MMP2‐responsive degradation	Rat full‐thickness calvarial defect (5 mm)	Highest BV/TV, with enhanced bone regeneration and anti‐inflammatory effect	[218]
Smart‐responsive composite hydrogel (photothermal‐responsive)	Photocrosslinked NIR/pH‐responsive network	Improved storage modulus and strength	Progressive degradation	Rat calvarial critical‐size defect	Improved trabecular regeneration and vascularized bone formation	[219]
Smart‐responsive composite hydrogel (pH‐responsive)	Dynamic Schiff‐base network	Optimized stiffness by nanoindentation	Acid‐triggered release	Rat calvarial critical‐size defect	MSC recruitment‐coupled angiogenic osteogenesis	[220]

## 5 Applications of Hydrogels for Bone Repair

**FIGURE 5:**
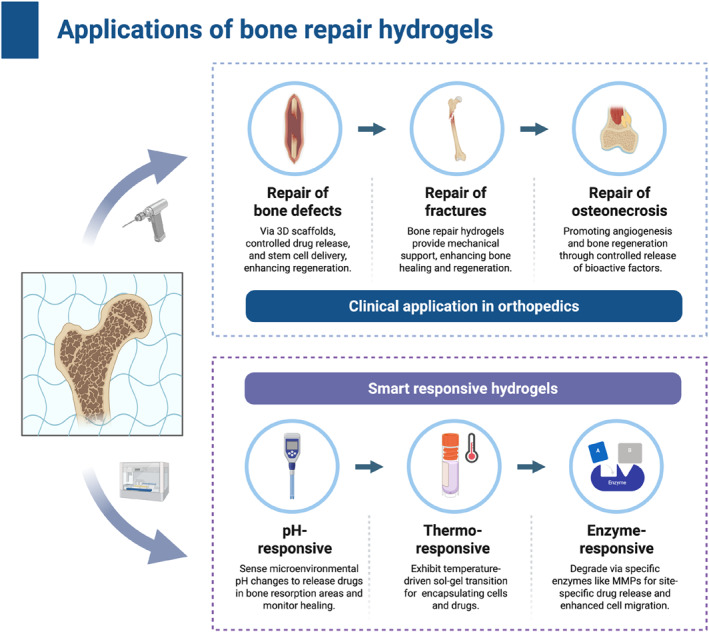
Applications of hydrogels for bone repair. From clinical orthopedic treatments to smart responsive hydrogel systems. Created with BioRender.com.

Hydrogels for bone repair exhibit stable biocompatibility, tunable physicochemical characteristics, and diverse functionalities. Their 3D network structure resembles the natural ECM, offering a supportive microenvironment that facilitates cell adhesion, proliferation, and differentiation. At the same time, this structure provides an efficient platform for loading bioactive molecules involved in bone regeneration. With the ongoing advances in materials science and regenerative medicine, hydrogels have evolved from simple space‐filling materials into intelligent systems that can respond to external stimuli, providing new approaches for managing complex bone defects (Figure 5).

### 5.1 Repair of Bone Defects

Bone defect repair is one of the main applications of hydrogels in bone regeneration. Large segment bone defects caused by trauma, tumor resection, or congenital malformation often show limited self‐healing ability, so biological materials are needed to support the regeneration process [211]. Due to its soft and adaptable 3D network, the hydrogel can closely fit irregularly shaped defects and form a supporting scaffold that is conducive to cell migration and proliferation. They can also carry a variety of biologically active factors, including BMP‐2 and VEGF, which can be continuously released, thus promoting osteogenic differentiation and angiogenesis [221]. Tan et al. used a BMP‐2‐loaded supramolecular hydrogel to promote periodontal bone regeneration. In a rat maxillary critical‐sized defect model, the hydrogel provided long‐term release of BMP‐2. After 8 weeks, the treated defects achieved a bone volume fraction of 56.7%, indicating enhanced periodontal bone regeneration and improved tissue architecture [222]. Wang et al. developed a scaffold‐based system that integrates silk fibroin and nHA to promote vascularized bone regeneration. By loading BMP‐2 and VEGF onto silk fibroin microspheres through a combination of covalent and physical interactions, sequential release of the growth factors was achieved. In a rat calvarial defect model, this system successfully promoted complete bone bridging using extremely low doses of cytokines [223].

Recent years have witnessed the development of various functionalized hydrogel systems for bone regeneration. GelMA‐based hydrogels, combined with nHA, for example, have demonstrated excellent osteogenic potential by enhancing mechanical strength while promoting mineralization through the release of calcium and phosphate ions [224, 225]. Wang et al. designed a composite hydrogel by incorporating mineralized nHA into GelMA. Their findings demonstrated that the biomimetic nanofibers improved the mechanical properties, biocompatibility, and osteogenic capacity of GelMA, with the 15% *m*‐HANFs/GelMA group showing the most suitable degradation rate and in vivo bone regeneration effectiveness [226]. In large animal studies, similar composite hydrogels have successfully repaired critical‐sized mandibular defects. Histological analysis shows that the newborn bone is seamlessly integrated with the host tissue, accompanied by a dense vascular network. In the sheep critical size defect model, Ingalve et al. proved that this complex hydrogel significantly increased vascular density, bone formation and new bone volume during the 12‐week implantation period [227]. Another progress is the development of self‐healing hydrogel systems. These materials rely on dynamic covalent interaction or supramolecular binding, which enables the hydrogel network to recover after structural destruction. This ability is especially beneficial for minimally invasive surgery that requires injection [228]. In addition to chemical cross‐linked hydrogels, physical cross ‐ linked hydrogels an important role in bone defect repair. Thermo‐responsive hydrogel based on PNIPAAm maintains a liquid state at room temperature, which is convenient for injection and transforms into a gel state under body temperature, thus providing a stable environment for cell survival and proliferation [229]. Zhang et al. designed a temperature‐responsive supramolecular hydrogel using a central poly (ethylene oxide) block and terminal PNIPAAm block with ureido pyrimidinone moieties randomly incorporated. This material forms a dynamic network, showing shear thinning and self‐healing characteristics, thus realizing the encapsulation and delivery of mesenchymal stem cells with high vitality. It represents a promising platform for cell‐based treatment of bone defect repair [230]. This intelligent hydrogel system can encapsulate stem cells and growth factors, form biologically active filling materials in the defect site, and accelerate the regeneration of bone tissue.

### 5.2 Repair of Fractures

Bone fracture repair is another application field of hydrogels. In traditional clinical practice, metal internal fixation devices are widely used to provide mechanical stability. However, they may cause complications, including stress occlusion, and may also require a second surgical removal device [231]. Hydrogels provide a biocompatible alternative or complementary treatment option because they can support fracture healing and actively participate in the bioremediation process [232]. In cases of simple fractures, injectable hydrogels show obvious advantages. Thermosensitive chitosan hydrogel undergoes sol‐to‐gel transformation under body temperature, so that it can fill irregular fracture gaps and act as a carrier for osteogenic agents (including parathyroid hormone). These systems help regulate the local microenvironment and accelerate the formation of bone scabs by controlling the release of bioactive factors [233]. Experimental research shows that hydrogels significantly promote fracture healing, and biomechanical tests further show that the maximum load‐bearing capacity and stiffness of fractures after treatment increase [234].

For more complex fractures, especially those accompanied by neurovascular damage, conductive hydrogels show considerable therapeutic prospects [235]. Liu et al. developed a piezoelectric conductive hydrogel scaffold. It can generate electrical stimulation through normal joint movement. This stimulus produces potential differences in promoting the migration of bone marrow mesenchymal stem cells. The upper layer of the bracket carries a positive charge that promotes cartilage differentiation, while the lower layer provides an anion signal that promotes osteogenic differentiation, so as to support the repair of integrated osteochondral through integrated mechanical and electrical signals [236]. Jing et al. also developed a photosensitive conductive hydrogel system (GelMA‐BP@Mg) for infectious bone defects. The hydrogel shows a strong antibacterial effect through the photothermal and photodynamic activity triggered by near‐infrared, and also improves the inflammatory microenvironment of the damaged site. The released conductive nanosheets and magnesium ions jointly stimulate the migration of Schwann cells and the extension of the process, which helps to rebuild the neural network related to bone repair [237].

Hydrogel also shows potential in the treatment of osteoporotic fractures. By adding antiresorptive agents, these systems can form a local drug trove at the fracture site, promote bone formation and inhibit bone resorption, so as to achieve double control of bone reconstruction [238]. Xiao et al. designed an intelligent composite hydroge, which has the ability of fast glue formation, injectability and self‐repair. The material releases stromal cell‐derived factor‐1 (SDF‐1) and calcitonin gene‐related peptide (CGRP) in a controlled manner. SDF‐1 enhances the recruitment of mesenchymal stem cells through the SDF‐1/CXCR4 signal axis to promote the formation of early bone scabs, while CGRP supports later mineralization and remodeling. Immunofluorescence analysis confirms that this hydrogel promotes neurovascular regeneration during fracture healing [239]. Other studies have developed injectable adhesive hydrogels that combine magnesium‐alendronate metal‐organic frameworks in gelatin and starch networks. This composite material is firmly attached to the irregular bone surface and gradually degrades in the acidic environment associated with osteoporosis, releasing Mg^2+^ that contributes to bone regeneration. In addition, the alendronate component reduces bone resorption and fibroblast activation‐related fibrosis by competitively binding osteosclerotic protein and regulating the Sclerostin/TGF‐β signaling pathway [240].

### 5.3 Repair of Osteonecrosis

Osteonecrosis is a difficult and often refractory orthopedic condition, mainly resulting from disrupted blood supply that ultimately leads to the death of bone tissue [241]. Because the pathogenesis of osteonecrosis is closely associated with vascular injury, successful repair depends not only on new bone formation but also on timely revascularization of the necrotic region. Therefore, hydrogel‐based therapeutic strategies for osteonecrosis should emphasize angiogenic‐osteogenic coupling rather than osteogenesis alone. In the context of vascular reconstruction, hydrogels carrying angiogenic factors have shown considerable potential. Heparinized hydrogels can bind growth factors, including VEGF, through electrostatic interactions while preserving their bioactivity [242, 243]. In animal models of femoral head necrosis, hydrogels incorporating angiogenic factors improve local blood perfusion, promote neovascularization, and slow the progression of necrosis. An illustrative example comes from a study using an alginate and HAP hydrogel loaded with graphene oxide‐based miR‐7b nanocarriers (GPC@miR). Through controlled release of GPC@miR, the hydrogel suppressed the osteoclast fusion protein DC‐STAMP, thereby reducing the formation of bone‐resorbing osteoclasts while preserving vessel‐associated osteoclast subsets. These vascular‐related osteoclasts then promote PDGF‐BB and VEGF‐A signaling, thus promoting angiogenesis and supporting bone regeneration [244].

The sequential release strategy has also attracted attention. The designed hydrogel can quickly release angiogenic factors in the early stage, followed by the continuous release of osteogenic factors, showing a better therapeutic effect than single‐factor hydrogel in the osteonecrosis model. Wang et al. developed PLGA and poly (D, L‐lactide) (PDLLA) nuclear shell microspheres, encapsulated VEGF and BMP‐2. The PDLLA shell can release VEGF early to promote angiogenesis, while the PLGA core provides continuous BMP‐2 release to support subsequent osteophytic differentiation. This delivery system significantly enhances CD31‐positive vascular infiltration and bone formation in the cranial defect model [245]. Similar concept has guided the development of the space‐time gradient hydrogel system. These systems initiate early angiogenesis by accelerating GelMA degradation and dimethyloxallyl glycine (DMOG) release, thus promoting the establishment of anterior vascular networks. The continuous degradation of silk protein then releases nHA to support osteopathic maturity. This method achieves functional vascularization in the cranial defect model and significantly enhances bone regeneration [246].

Another promising strategy is to combine hydrogel with bioactive ceramics (including HAP and bioglass). HAP can provide an inorganic phase that simulates bones, participate in the enrichment and anchoring of growth factors, and improve the porous and surface microenvironment, thus helping the hydrogel system to locally retain these regeneration signals in the defect site and promote angiogenesis and bone repair [247, 248]. In contrast, bioglass helps to enhance the bioactive mineral microenvironment [249]. Through appropriate bioactive ion doping, bioglass can form an immune microenvironment that supports the time coordination of angiogenesis and tissue repair, thus promoting vascular bone regeneration [34, 250].

### 5.4 Regulation of the Immune Microenvironment

Immune regulation has become the core design principle of hydrogels for bone repair, and a well‐coordinated immune microenvironment is the key to successful tissue regeneration [251, 252]. In immune cells, macrophages are particularly important, because their phenotypic transformation seriously affects cytokine secretion, angiogenesis, mesenchymal stem cell collection and osteogenesis, and matrix remodeling. It is worth noting that bone healing requires a temporarily coordinated immune response and not just inhibiting inflammation. Early pro‐inflammatory macrophage activity is necessary for fragment removal and initiation of repair signal conduction, and the subsequent transformation of the regenerative phenotype is necessary for the regression of inflammation and the progression of vascular bone formation. Therefore, hydrogel systems are increasingly designed to not only directly promote bone formation, but also regulate the behavior of macrophages and reshape the immune microenvironment through space‐time control.

Current immunomodulatory hydrogel strategies include the delivery of anti‐inflammatory agents, the incorporation of bioactive ions or nanomaterials, and the use of responsive matrices that modulate local cytokine signaling. Anti‐inflammatory hydrogels functionalized by drugs such as dexamethasone have been reported to affect the behavior of macrophages. For example, silk protein hydrogels loaded with dexamethasone can promote macrophage polarization toward an M2 phenotype, thus reducing excessive inflammatory reactions [253]. This transition to an anti‐inflammatory and regenerative environment not only creates favorable conditions for tissue repair, but also enhances the osteogenic differentiation of mesenchymal stem cells. In addition to the traditional anti‐inflammatory factor delivery, more complex hydrogel systems have also been developed to regulate the immune microenvironment through intercellular communication and metabolic support. For example, an engineering graded hydrogel with immunoreactivity has been proven to mediate the targeted mitochondrial transfer from macrophages to bone marrow mesenchymal stem cells, thus restoring BMSCs bioenergetics damaged by the local inflammatory environment and ultimately promoting bone regeneration [254].

### 5.5 Smart Responsive Hydrogels

Intelligent responsive hydrogel is a unique biological material that can actively interact with the bone healing microenvironment. Unlike traditional hydrogels, they cannot simply fill in defects or serve as delivery carriers. On the contrary, they can react to local or external stimuli and then change their behavior in a useful way, such as gelation, degradation, drug release, mechanical regulation or signal presentation. In bone repair, smart behavior is not just injectable or self‐healing. This mainly depends on whether the hydrogel can respond reliably to biologically related signals and regulate the local repair environment. This difference is important because many hydrogels have processing or delivery functions, while only some hydrogels can really respond to stimulation in a way related to bone repair. According to the source of stimulation, intelligent hydrogels used for bone repair can be divided into two major categories. The endogenous reaction system is triggered by local biochemical changes, such as pH, reactive oxygen species (ROS), or enzyme activity. The exogenous response system responds to external triggers (e.g., temperature, light or mechanical stimulation). This classification helps to distinguish between systems that mainly improve processing and transportation and those that directly regulate bone repair.

In the endogenous response system, pH‐response hydrogel is one of the most widely studied systems. Their theoretical basis is that the microenvironment of bone damage is not chemically static [255] Physiological bone tissue is maintained within a narrow pH range, and inflammatory reactions, osteoblast‐mediated bone resorption, infection, or ischemic microenvironment often lead to local acidification [256, 257].

The hydrogel combined with the ionizing group can respond to these changes and cause swelling, swelling or structural loosening, so that the therapeutic agent can be locally released in the part with the highest biological needs. For example, in osteoporotic bone repair, polymers containing tertiary amine groups, such as poly(dimethylaminoethyl methacrylate), can be protonated in acidic regions, causing network swelling and anti‐bone aspiration. The site‐specific release of the receiving agent [258, 259]. Animal studies of osteoporosis rat models show that this strategy can improve the bioavailability of local drugs while reducing systemic adverse reactions [260]. A pH‐responsive hydrogel can be designed to monitor bone healing in real time. By incorporating pH‐sensitive fluorescent components into the hydrogel network, dynamic pH fluctuations can be visualized as a real‐time indicator of repair progress [261, 262]. This self‐reporting ability provides a basis for personalized treatment and guides treatment decision‐making through in situ monitoring [263]. In bone defects, several processes including inflammation, osteoblast activation, ischemia and infection may lead to local acidification.

ROS‐responsive hydrogel constitutes another important category of endogenous responsive intelligence systems, which should be clearly emphasized, because oxidative stress is a common feature of inflammatory bone defects, osteoporotic microenvironment, diabetic bone healing and implant‐related tissue damage. In these cases, excessive ROS not only reflects tissue stress, but also actively damages the function of osteoblasts, stem cell survival and matrix regeneration. Therefore, ROS‐responsive hydrogels are attractive because they can combine oxidative stress perception with antioxidant buffering or therapeutic release. For example, it has been reported that the Mg@PEG‐PLGA hydrogel system can be injected to release hydrogen and magnesium ions during degradation, thus removing ROS, regulating inflammation, and promoting bone regeneration of osteoporotic defects [93]. Compared with pH‐responsive hydrogels, ROS‐responsive hydrogels can better reflect the severity of inflammation or oxidative damage. However, this strategy also faces conversion challenges, because ROS levels vary greatly between different disease states, organizations and healing stages, and the threshold required for reliable in vivo triggers is still not standardized enough.

Thermo‐responsive hydrogel constitutes another widely studied intelligent system. Its unique sol‐gel transformation makes it particularly suitable for minimally invasive operation [264]. These materials usually contain hydrophobic units, such as isopropyl in PNIPAAm, which are dehydrated and contracted as the temperature rises, thus forming a gel [265]. This characteristic is especially beneficial to cell delivery. PNIPAAm‐based hydrogel remains liquid at room temperature, can be incorporated evenly with cells, and converted into gel after injection into the body, so as to fix the delivered cells at the defect site [266]. The study shows that this package improves cell viability, improves fixation, and supports effective bone regeneration [267]. In order to further optimize clinical efficacy, some systems have added biodegradable components to ensure that the hydrogel gradually degrades after completing the therapeutic function, so that the new tissue grows into the defect space [268]. In drug delivery applications, thermo‐responsive hydrogels can also precisely control the release curve [265]. Some advanced systems are realized by integrating gold nanorods or similar photothermal conversion materials, which can respond to near‐infrared irradiation and realize remote‐controlled drug release [269]. These designs show special prospects in deep tissue repair, such as vertebral bone reconstruction after vertebroplasty. In vivo experiments show that intermittent near‐infrared stimulation can produce a pulse release mode closer to physiological secretion dynamics [270]. Although thermo‐responsive hydrogels have advantages in terms of operation, injectability and space retention, their responsiveness is often not closely related to the endogenous responsive system with the biological changes in bone repair.

Enzyme‐responsive hydrogels are specially designed to respond to specific enzyme activity, with high precision and selectivity, which is very consistent with the principles of precision medicine [271]. In the process of bone repair, the expression level of matrix metalloproteinase (MMPs), ALP and other enzymes changes dynamically. Therefore, these enzymes can be used as biological triggers for responsive hydrogel systems. MMPs‐responsive hydrogel is one of the most widely explored hydrogels. MMPs play an important role in ECM remodeling, and their expression levels are closely related to tissue repair activity [272]. By using MMPs‐sensitive peptide sequences as cross‐linked elements, hydrogels can be programmed to be selectively degraded in areas with increased MMP activity. This process promotes the release of local drugs and creates channels to promote cell migration and tissue implantation [273]. In the defect model of critical size, MMPs‐responsive hydrogel shows better integration and regeneration effects than traditional materials [274]. The ALP‐responsive hydrogel emphasizes the mineralization stage of bone repair. ALP acts as an early marker of osteogenic differentiation by hydrolyzing phosphate esters into inorganic phosphate ions, which are essential for mineral deposition [275]. Some hydrogels incorporate ALP‐sensitive functional groups such as phosphotyrosine. After hydrolysis by ALP, these groups generate tyrosine residues that promote cell adhesion and osteogenic differentiation [276]. This enzymatic response simultaneously elevates local phosphate concentrations, thereby promoting apatite deposition and matrix mineralization. Compared with pH‐ and thermo‐responsive systems, enzyme‐responsive hydrogels are more closely coupled to the biological events of bone healing. However, their effects in vivo still depend on how enzyme activity varies across different phases and regions of the repair process.

**FIGURE 6:**
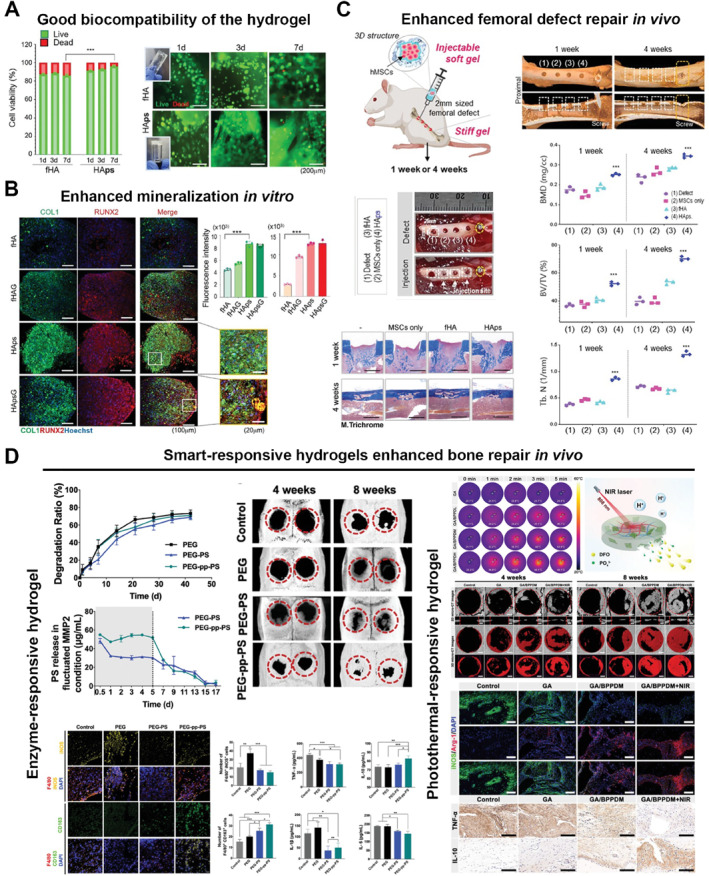
Representative literature examples of conventional and smart‐responsive hydrogels for bone repair. (A) Representative live/dead staining and cell viability results showing the good biocompatibility of the hydrogel. (B) Representative osteogenic marker staining demonstrating enhanced mineralization in vitro. (C) Representative in vivo outcomes, including gross observation, micro‐computed tomography, histological staining, and quantitative analysis, showing enhanced femoral defect repair. (D) Representative literature examples of smart‐responsive hydrogels promote bone repair in vivo through enzyme‐responsive or photothermal‐responsive mechanisms and modulation of the regenerative microenvironment. Adapted under terms of the CC‐BY license [213, 218, 219]. Copyright 2024, The Authors, published by John Wiley and Sons.

Mechanoresponsive hydrogels are also relevant to bone repair because skeletal tissue is inherently load‐bearing and mechanosensitive. However, this area remains less developed than other responsive strategies [277, 278]. Overall, smart responsive hydrogels enable more dynamic interactions with the repair microenvironment than conventional scaffolds. As shown in Figure 6, representative literature examples indicate that hydrogels can support bone repair through good biocompatibility, enhanced mineralization, and improved defect healing, whereas emerging smart‐responsive hydrogels can further promote bone regeneration by exerting stimulus‐responsive functions and more refined regulation under specific conditions [213, 218, 219]. Current studies suggest that pH‐ and ROS‐responsive systems are particularly effective under inflammatory or oxidative conditions, thermo‐responsive hydrogels are mainly used for injectable delivery and controlled release, and enzyme‐responsive hydrogels are more directly involved in matrix remodeling and mineralization. Further progress will depend on improved trigger specificity, reproducibility, and in vivo validation.

## 6 Challenges and Prospects

### 6.1 Major Challenges in Current Research

Before hydrogel for bone repair is widely used in conventional clinical practice, there are still many challenges to be solved. These challenges come not only from the inherent limitations of current material design and functional performance, but also from practical obstacles related to clinical transformation, emphasizing the need for a clearer and more systematic development strategy.

Biosafety is the basic premise of clinical application. Although many hydrogels are based on materials with recognized biocompatibility, the inclusion of crosslinkers, growth factors, nanoparticles or other functional additives may cause additional safety issues [279]. If the residual reagent is not completely removed during the purification, chemical cross‐linking agents such as glutaraldehyde can have cytotoxic effects. There are still limitations in terms of mechanical properties, especially for weight‐bearing bone repair [280]. Most hydrogels do not have enough mechanical strength to withstand physiological pressure. Although the enhancement methods of nHA, GO or other inorganic phases improve these properties, it is still difficult to achieve the balance between elasticity and rigidity.

Sterilization compatibility is a key design limitation, because hydrogel systems containing dynamic bonds, photoactive groups, proteins, extracellular vesicles or living cells may not be able to withstand conventional terminal sterilization, and may require sterile production processes, which greatly increases its complexity [114]. Large‐scale production cannot be inferred from benchtop gelation alone; Successful transformation requires raw material standardization, GMP compatibility, preferably a closed or automated production system, and robust process control to maintain rheology, gel dynamics, injectability and cross‐batch and operator biological activity [281]. The feasibility of regulation should be considered from the beginning, because hydrogel platforms can be classified differently according to their main use as structural biological materials, drug delivery systems or carriers of cells and other biologically active ingredients. Therefore, the clinical indications of material composition, manufacturing strategy, quality control and expected must be coordinated in the early stage of development [282].

Storage stability is still an obstacle to be developed. If the hydrogel cannot maintain the key physicochemical and biological properties during freezing, freeze‐drying or transportation, the hydrogel that performs well immediately after preparation may still fail in the conversion process, and the preparation with monthly stability can better reflect the real spot potential [283]. Reproducibility should be regarded as the transformation endpoint of the core. Recent guidelines emphasize purpose‐friendly quality standards, standardized workflows and unified Characterization methods to minimize differences between batches and sites, which are particularly critical for personalized or bio‐prepared hydrogel construction [284].

### 6.2 Prospects for the Application of Hydrogels in Bone Repair

The future development of hydrogels for bone repair may focus on establishing a clear clinical transformation path, creating a reliable real‐time monitoring system, and improving large‐scale manufacturing to improve accessibility.

#### 6.2.1 Clinical Translation and Regulatory Approval

Hydrogel for bone repair must meet the strict standards of safety, stability and therapeutic effect. Increased cooperation between researchers, clinicians and regulators helps to simplify assessment and approval procedures. The previous successful approval of hydrogel‐based wound dressings and injectable cartilage repair systems provides a useful basis. In the future, the establishment of regulatory guidelines for hydrogel bone repair materials will greatly accelerate its clinical transformation.

#### 6.2.2 Long‐Term Performance and Monitoring

Long‐term in vivo performance is crucial for ensuring safe and effective clinical use. Hydrogels for repair must avoid causing persistent inflammation or producing harmful degradation products. Imaging‐guided monitoring progress (including hydrogel integrated with MRI contrast agent) can now visualize hydrogel degradation and bone healing in real time. These technologies provide valuable information about long‐term performance. In addition, the development of self‐monitoring hydrogels that can report changes in the microenvironment may support more personalized treatment strategies.

#### 6.2.3 Market Adoption and Accessibility

The production of hydrogels for bone repair must be cost‐effective and clinically accessible. It is necessary to reduce manufacturing costs and establish a scalable production process. The successful commercialization of hydrogel products in wound care shows that similar methods are feasible in bone repair. Strengthening cooperation between industry, academia and clinical institutions is of great significance to reduce costs, improve medical insurance reimbursement, and promote the wider application of advanced hydrogel technology in clinical practice.

## 7 Conclusion

Hydrogel has the characteristics of simulating ECM, adjustable mechanical properties and good biocompatibility, and is an ideal platform for bone repair. This review systematically outlines the construction strategy of hydrogels, emphasizing the synergy between material selection, precise cross‐linking and functional modification of bioactive molecules, nanomaterials or cells. Advanced applications are not only limited to structural support, but also include intelligence, stimulus response system and active immune regulation. Despite remarkable preclinical success in the treatment of bone defects, fractures and osteonecrosis, challenges in transformation still exist, including optimizing mechanical strength, ensuring precise degradation dynamics and ensuring long‐term biosafety. Future research must give priority to overcome these obstacles and accelerate the clinical application of hydrogel‐based therapy through multidisciplinary cooperation and standardized evaluation paths.

## Author Contributions

**Miaomiao Wang:** conceptualization, methodology, formal analysis, investigation, writing – original draft. **Qianwei Su:** conceptualization, methodology, formal analysis, investigation, writing – original draft. **Yuezhou Wu:** conceptualization, methodology, formal analysis, investigation, writing – original draft. **Xiaoyi Liang:** conceptualization, methodology, formal analysis, investigation, writing – original draft. **Fengjin Zhou:** conceptualization, formal analysis, writing – review and editing. **Zhanghua Li:** investigation, writing – review and editing. **Yingying Jing:** writing – review and editing, supervision, funding acquisition. **Jiacan Su:** writing – review and editing, supervision, funding acquisition.

## Conflicts of Interest

The authors declare no conflicts of interest.

## Data Availability

The data that support the findings of this study are available from the corresponding author upon reasonable request.
